# A Novel Biocontainment Strategy Makes Bacterial Growth and Survival Dependent on Phosphite

**DOI:** 10.1038/srep44748

**Published:** 2017-03-20

**Authors:** Ryuichi Hirota, Kenji Abe, Zen-ichiro Katsuura, Reiji Noguchi, Shigeaki Moribe, Kei Motomura, Takenori Ishida, Maxym Alexandrov, Hisakage Funabashi, Takeshi Ikeda, Akio Kuroda

**Affiliations:** 1 Department of Molecular Biotechnology, Graduate School of Advanced Sciences of Matter, Hiroshima University, Higashi-Hiroshima, Hiroshima 739-8530, Japan; 2 Process Development Laboratories, Research Institute for Bioscience Products & Fine Chemicals, Ajinomoto Co., Inc., 1-1 Suzuki-cho, Kawasaki-ku, Kawasaki-shi, Kanagawa 210-8681, Japan

## Abstract

There is a growing demand to develop biocontainment strategies that prevent unintended proliferation of genetically modified organisms in the open environment. We found that the hypophosphite (H_3_PO_2_, HPt) transporter HtxBCDE from *Pseudomonas stutzeri* WM88 was also capable of transporting phosphite (H_3_PO_3_, Pt) but not phosphate (H_3_PO_4_, Pi), suggesting the potential for engineering a Pt/HPt-dependent bacterial strain as a biocontainment strategy. We disrupted all Pi and organic Pi transporters in an *Escherichia coli* strain expressing HtxABCDE and a Pt dehydrogenase, leaving Pt/HPt uptake and oxidation as the only means to obtain Pi. Challenge on non-permissive growth medium revealed that no escape mutants appeared for at least 21 days with a detection limit of 1.94 × 10^−13^ per colony forming unit. This represents, to the best of our knowledge, the lowest escape frequency among reported strategies. Since Pt/HPt are ecologically rare and not available in amounts sufficient for the growth of the Pt/HPt-dependent bacteria, this strategy offers a reliable and practical method for biocontainment.

Genetically modified microorganisms (GMMs) play a vital role in the chemical, pharmaceutical, and food industries. Owing to recent developments in the fields of synthetic biology^1^,^2^ and genomics^3^,^4^, there is a growing demand for functionalized GMMs that can be safely used outside of enclosed laboratory facilities. Novel applications of GMMs include the production of biofuels, bioremediation^5^, and clinical treatments^6^. One of the biggest concerns for the practical use of GMMs in the open environment is the possibility of uncontrolled proliferation that could, in a worst-case scenario, endanger public health or biodiversity. Therefore, strategies for safeguarding against the spread and proliferation of GMMs in the environment must be developed in order to realize their practical applications.

Biocontainment is a methodology that involves the design of GMMs that are only able to grow and survive under particular conditions^7^. Current biocontainment strategies can be classified into active and passive forms. The former employ killing switches such as toxin/antitoxin genes^5^,^8^ and self-destructing DNA^9^, while the latter create requirements for externally supplied molecules (auxotrophy) by eliminating essential gene functions^10^,^11^. Several auxotrophic strategies that render cells dependent on artificial compounds have recently been reported^12^,^13^,^14^. Two research groups developed genomically recoded *Escherichia coli* strains in which expanded codons for non-standard amino acids (NSAAs) were introduced into essential genes and demonstrated that the resultant strains strictly rely on the availability of NSAAs for their growth. Lopez and Anderson^12^ developed an alternative biocontainment strategy that introduces ligand-dependent essential genes into an *E. coli* BL-21 strain. Only the addition of the synthetic chemical benzothiazole in the culture medium rescues bacterial growth by restoring the activities of the essential gene products. Due to the absence of these synthetic chemicals in nature, such microbes should be unable to grow in the open environment.

One major concern for biocontainment strategies based on nutrient auxotrophy is the emergence of escape mutants that no longer rely on the required chemicals. A common mechanism of generating escape mutants is spontaneous genetic mutations that circumvent artificially introduced functional defects. In the abovementioned works involving genomically recorded *E. coli*, the introduction of NSAA codons into various essential genes resulted in escape frequencies of approximately 10^−4^ to 10^−7^ escape mutants per colony forming unit (CFU)^13^,^14^ depending on the modified gene. In order to reduce the frequency of escape mutant generation, one can increase the number of alleles containing the NSAA codons or ligand-dependent genes^13^,^14^. In addition, it is possible to introduce multiple safeguard systems based on different containment strategies^13^,^14^,^15^. However, to completely suppress the generation of escape mutants, researchers had to add several genetic modifications that impede escape mutations or introduce an additional containment strategy based on diaminopimelic acid auxotrophy^16^. The resultant containment systems achieved an undetectable number of escape mutants with an assay detection limit of less than 6.3 × 10^−12^ per CFU^13^,^14^. Thus, the development of novel containment strategies with low escape frequencies and high compatibility with other strategies is important for achieving impregnable biocontainment.

Phosphorus (P), as a component of nucleic acids, lipids, and various cellular metabolites, is an essential nutrient for all living organisms. Nearly all biologically available P in the environment is in the form of phosphate (H_3_PO_4_, Pi) and its esters, in which the P valence is +5. Several groups of bacteria also possess an oxidization system for oxidizing P compounds such as phosphite (H_3_PO_3_, Pt: P valence +3) and, less commonly, hypophosphite (H_3_PO_2_, HPt: +1). The molecular basis of Pt/HPt-oxidation in bacteria was established using the soil bacterium *Pseudomonas stutzeri* WM88^17^. In this bacterium, HPt is oxidized to Pt by HPt dioxygenase (HtxA)^18^, and the resultant Pt is oxidized to Pi by NAD-dependent Pt dehydrogenase (PtxD)^19^. Recent evidences mainly obtained from the marine and soil environment suggested the presence of biological P redox cycle in nature^20^,^21^,^22^,^23^. However, in contrast to organic reduced P compounds such as phosphonates (+3)^23^,^24^, Pt and HPt are rarely detected in the environment^24^,^25^.

The mechanisms involved in the metabolism of reduced P compounds are potentially useful for developing novel biotechnology applications^26^,^27^. Our previous work demonstrated that PtxD can be used as a selectable marker in yeasts^26^. Since Pt cannot be metabolized without oxidation to Pi, the introduction of *ptxD* into cells that are incapable of utilizing Pt confers the ability to grow on medium containing Pt as the sole source of P. Based on this expanded Pt utilization ability of a host organism and given the scarcity of Pt in the environment, we conceived that engineered dependency on Pt could also be used as a strategy for biocontainment. In this scenario, if a strain expressing PtxD and a Pt transporter were engineered to be unable to take up Pi or other Pi compounds commonly present in the environment, it would be unable to grow without exogenous Pt (Fig. 1a). This strategy, however, would require a Pt-specific transporter that is unable to transport Pi.

In this study, we found that the HPt transporter HtxBCDE of *P. stutzeri* WM88 exclusively takes up Pt and HPt but not Pi. This finding enabled us to develop a biocontainment strategy utilizing Pt or HPt as a required nutrient. Considering the extremely low escape frequency and the simplicity of required genetic modifications, this strategy may contribute to the development of a reliable and cost-effective biocontainment system for practical applications. This is the first report of the development of a biocontainment strategy based on the metabolism of P, a linchpin molecule of cellular metabolism.

## Results

### The HPt transporter HtxBCDE takes up Pt but not Pi

**Figure 1:**
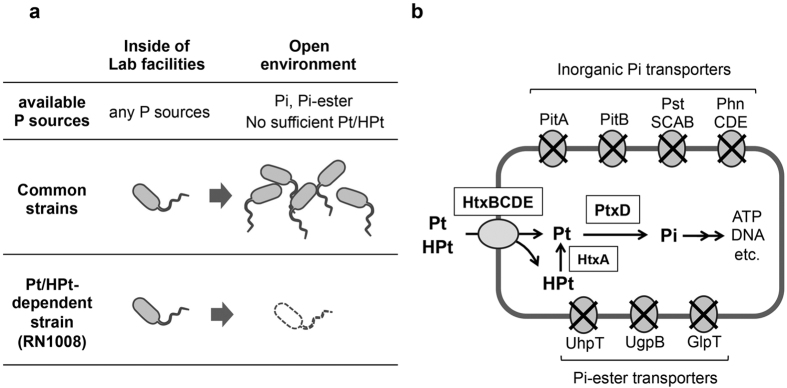
Creation of engineered dependency on Pt/HPt. (**a**) Concept for the biocontainment strategy using engineered dependency on Pt/HPt. Under laboratory conditions, microbial growth is maintained by using any P source, including Pt and HPt. Common microbial strains are able to grow both inside and outside of laboratory facilities by using Pi or organic Pi compounds as P sources. A Pt/HPt-dependent strain, which can utilize Pt or HPt but not Pi, is not able to grow unless Pt/HPt is provided as a P source under laboratory conditions. This Pt/HPt-dependent strain is not able to grow in the environment because Pt and HPt are ecologically rare and not available in amounts sufficient to support bacterial growth. (**b**) Schematic of the engineered P metabolic pathway for biocontainment. Dependency on Pt or HPt is created by disruption of endogenous Pi and organic Pi transporters and exogenous expression of HtxBCDE and PtxD. HtxBCDE takes up Pt/HPt but not Pi or organic Pi compounds. PtxD and HtxA expression confer Pt- and HPt-oxidation activities, respectively.

**Figure 2:**
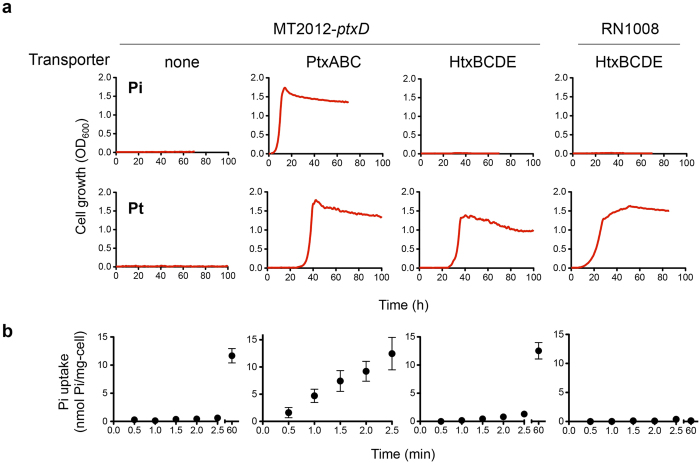
Characterization of the Pi and Pt transport abilities of Ptx and Htx transporters. (**a**) Growth of MT2012-*ptxD* expressing PtxABC or HtxABCDE on MOPS-Pi (top) or MOPS-Pt (bottom). MT2012-*ptxD* was transformed with a pMW118-based transporter expression plasmid. Cell growth was monitored every hour by measuring cell turbidity at 600 nm using an OD monitor. The data are representative of two independent experiments with essentially the same results. (**b**) [^32^P]Pi uptake of the strains. The values expressed as mean ± s.d. of three biological replicates.

The key requirement for re-engineering the P metabolic system is a transporter that takes up Pt but not Pi (Fig. 1b). We expected that the Pt transporter, PtxABC, would satisfy this requirement. Therefore, we first investigated the substrate specificity of PtxABC. The MT2012^28^, an *E. coli* strain lacking all indigenous Pi transporters (PitA, PitB, PstSCAB, and PhnCDE), can be used as a host strain to investigate Pi transport abilities of heterologously expressed transporters. Since MT2012 is also unable to grow on Pt (Fig. 2a), we constructed MT2012-*ptxD* to evaluate both Pi- and Pt-specific activities of PtxABC, and found that the resultant strain could grow on both MOPS-Pi and MOPS-Pt. This result indicated that, unfortunately, PtxABC can transport both Pi and Pt (Fig. 2a). We then tested HtxBCDE, a transporter for a more reduced form of inorganic P, HPt. The expression of HtxABCDE from *P. stutzeri* WM88^17^ enabled MT2012-*ptxD* to grow on MOPS-Pt but not on MOPS-Pi (Fig. 2a), indicating that HtxBCDE transports only the reduced form of inorganic P.

To confirm that HtxBCDE is really unable to take up Pi, we performed Pi uptake analysis using [^32^P]Pi. Consistent with the results of the growth assay, almost no Pi uptake was observed in MT2012-*ptxD* expressing HtxABCDE as well as in the control strain, confirming that HtxBCDE is not able to import Pi. In contrast, MT2012-*ptxD* expressing PtxABC took up Pi at the rate of approximately 5.4 ± 1.1 nmol Pi/min/mg cell (dry cell weight) (*n* = 3, mean ± s.d.) (Fig. 2b). It should be noted that the prolonged incubation (60 min) of MT2012-*ptxD* and MT2012-*ptxD* expressing HtxABCDE resulted in increased radioactivities (approximately 0.2 nmol Pi/min/mg cell). This may be due to the incorporation of trace amounts of Pi via organic Pi transporters (GlpT, UgpG, and UhpT), although such an unspecific Pi uptake is insufficient to support cell growth^29^. Thus, these results together indicated that HtxBCDE has the required characteristics for engineering a reduced-P based metabolic system. A novel combination of HPt transporter and Pt dehydrogenase (PtxD) was therefore used instead of PtxABC in our biocontainment strategy (Fig. 1). Since MT2012-*ptxD* expressing HtxABCDE was also able to grow on MOPS-HPt (see below), co-expression of HtxA additionally confers HPt-oxidation activities. Therefore, HPt can also be used as a required nutrient.

### Engineering of an *E. coli* strain dependent on Pt/HPt for growth

**Figure 3:**
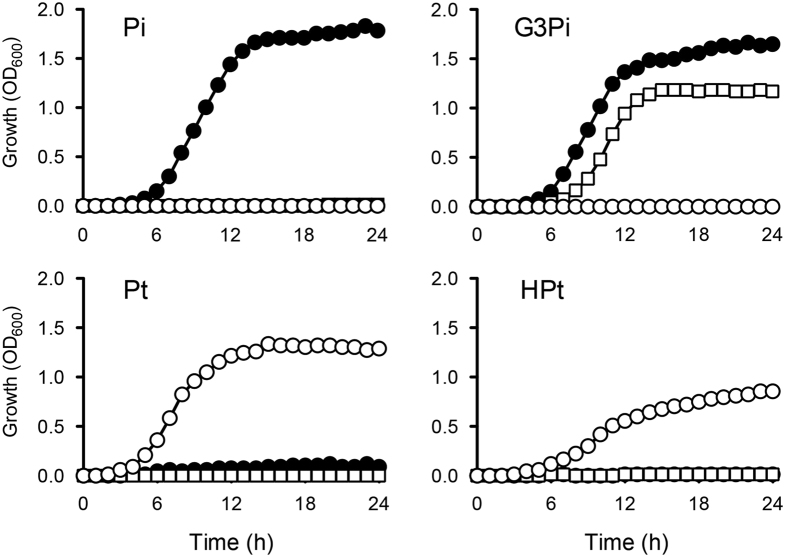
Growth of *E. coli* strains on MOPS media containing four different P sources. Growth of *E. coli* MG1655 (closed circles), MT2012 (open squares), and RN1008 (open circles) on MOPS media containing 1.0 mM Pi, G3Pi, Pt, or HPt. Optical densities at 600 nm were measured every hour. The data are representative of two independent experiments with essentially the same results.

As expected, MT2012-*ptxD* expressing HtxABCDE was unable to grow on MOPS-Pi (Fig. 2). However, since this strain still possesses three transporters for organic Pi, including GlpT, UgpB (for G3Pi), and UhpT (for hexose phosphate), it can grow in the presence of organic Pi (Fig. 3, open squares). In order to completely eliminate organic Pi transport and make the strain Pt/HPt-dependent, we created strain RN1008, which lacks the four transporter genes for Pi (*pitA, pitB, phnC*, and *pstSCAB*) and those for organic Pi (*glpT, ugpB*, and *uhpT*) and harbors Ptac4071-ptxD/pTWV229 and htxABCDE/pSTV28 (Fig. 1). This strain also lacks *phoA*, the gene for alkaline phosphatase, which can oxidize Pt in the periplasmic space. As expected, RN1008 was only able to grow on MOPS-Pt or MOPS-HPt media, and not on MOPS-Pi or MOPS-G3Pi (Figs 2a and 3 open circles). Pi uptake analysis also showed that almost no Pi was taken up by RN1008, even after 60 min of incubation (Fig. 2b), confirming that the incorporation of trace amounts of Pi in MT2012-*ptxD* was via the organic Pi transporters. The growth rates of RN1008 on MOPS-Pt and MOPS-HPt were 0.40 ± 0.02/h and 0.31 ± 0.03/h (*n* = 3, mean ± s.d.), respectively, which are 93% and 71%, respectively, of that of wild-type (MG1655) cultured in MOPS-Pi medium. The slower growth in MOPS-HPt medium could be due to the requirement for 2-oxoglutarate as a cofactor in HPt oxidation by HtxA^18^. Thus, Pt is the preferred selective nutrient for growing RN1008.

### Growth and environmental challenge of the Pt/HPt-dependent strain

**Figure 4:**
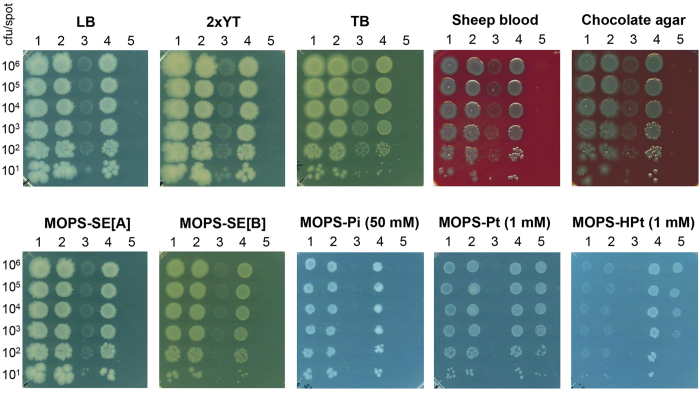
Growth of RN1008 on diverse types of media plates. Spot assay to assess growth of RN1008 and related strains on various types of solid media. MOPS-SE were prepared from two types of humic soils to create MOPS-SE[A] and MOPS-SE[B]. Pictures were taken at 48 h after incubation. Strains used (left to right): MG1655, MG1655 harboring Ptac4071-ptxD/pTWV, MT2012, RN1007, and RN1008.

To investigate the possibility that RN1008 is able to grow using other Pi compounds, cells were challenged to diverse types of media. A spot assay was carried out with various types of solid media plates. This revealed that RN1008 was unable to form colonies on LB, 2xYT, and Terrific Broth plates for at least seven days (Fig. 4). Two types of enriched blood agar media, including sheep blood and chocolate agar media, also failed to support the growth of RN1008, suggesting that none of the biological P compounds in these media were able to support the growth of RN1008 (Fig. 4). To investigate the effect of P compounds present in the environment, we prepared two MOPS media containing soil extract (SE). The total P contents of MOPS-SE[A] and -SE[B] were approximately 1.99 mM and 0.36 mM in which 1.60 mM and 0.04 mM of P were present as inorganic Pi, respectively. These MOPS-SE media also failed to support the growth of RN1008 (Fig. 4), suggesting that environmentally available P compounds cannot support the growth of RN1008. Phosphonates, a group of organophosphorus compounds that have direct carbon-phosphorus (C-P) bonds, are reduced P compounds produced mainly by Actinobacteria^30^. We examined the growth of RN1008 in MOPS medium containing 1.0 mM methylphosphonate, ethylphosphonate, or aminoethylphosphonate and found that none of these were able to serve as a P source for RN1008 (data not shown). These results strongly suggest that RN1008 is unable to grow in the open environment; therefore, the engineered Pt/HPt dependency represents a novel strategy for biocontainment.

### Pt/HPt-dependent strain does not yield escape mutants under non-permissive growth conditions

**Figure 5:**
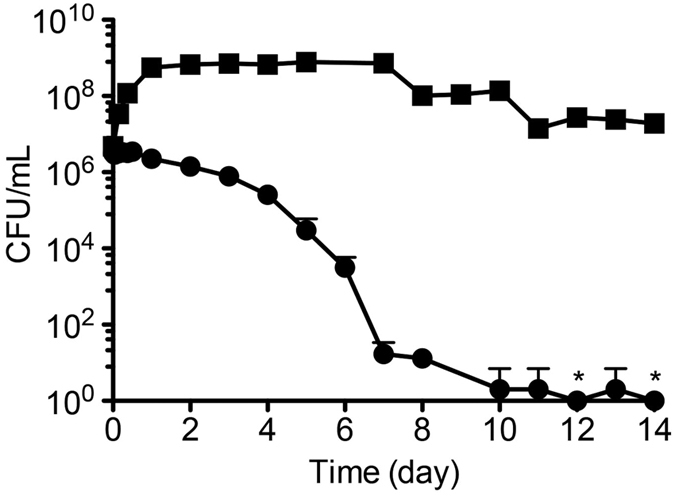
Long-term stability of RN1008 in liquid media. A culture of approximately 10^8^ RN1008 cells grown in MOPS-Pt medium was challenged over 14 days by growth on either permissive (MOPS-Pt, square symbols) or non-permissive (2xYT, circle symbols) media. Asterisks denote no CFU were observed. The values are expressed as mean + s.d. of three technical replicates. The data are representative of two biological replicates with essentially the same results.

Next, in order to assess the reliability of this containment strategy, we investigated the frequency of escape mutant generation under non-permissive growth conditions. Approximately 5.2 × 10^12^ cells of RN1008 were incubated on non-permissive (2xYT) growth plates and did not form any colonies for 21 days, indicating that RN1008 does not yield any escape mutants with an assay detection limit of less than 1.9 × 10^−13^ per CFU. To investigate the viability of RN1008 during cultivation, approximately 10^8^ cells were added to 0.1 L of 2xYT (non-permissive medium) or MOPS-Pt (permissive medium) and cultured for 14 days. RN1008 was unable to form colonies on non-permissive medium plates (data not shown), demonstrating that RN1008 did not yield any escape mutants during long-term liquid culture. In terms of the viability of RN1008, CFU number quickly declined during cultivation in 2xYT liquid medium, dropping to less than 100 CFU (<1.0 × 10^−4^% of initial inoculum) at seven days and below the detection limit at 14 days after inoculation (Fig. 5). In contrast, the viability of RN1008 under permissive culture condition (MOPS-Pt) did not significantly decrease over 14 days (Fig. 5). These results indicate that the viability of RN1008 rapidly decreases in non-permissive culture conditions.

## Discussion

In this work, we developed a novel biocontainment strategy by engineering an *E. coli* strain that is dependent on Pt/HPt as required P sources. Engineering of Pt/HPt dependency required disruption of all endogenous P transporters supplemented by exogenous expression of PtxD and a P transport system that takes up Pt/HPt but not Pi. Initially, we expected that the Pt transporter, PtxABC, could be used as such a P transport system. However, we found that PtxABC also transports Pi. The finding that the HPt transporter HtxBCDE also takes up Pt but not Pi, enabled us to create an engineered Pt/HPt metabolic pathway for biocontainment. HtxBCDE, which is a crucial component of this biocontainment strategy, is a binding protein-dependent HPt transporter belonging to the ATP-binding cassette (ABC) transporter superfamily^31^. Within the HtxBCDE protein complex, discrimination between Pi and Pt/HPt is due to HtxB, a periplasmic substrate-binding protein (SBP). The molecular mechanism of Pi sequestration by SBPs has been elucidated in Pi binding protein PstS^32^,^33^. A Pi ion binds to PstS via 12 hydrogen bonds formed between the four oxygen atoms of a Pi molecule and -OH and -NH groups of the PstS protein. Eight conserved amino acid residues^32^ located at the substrate-binding cleft of the PstS protein are responsible for hydrogen bond formation. Currently, there is no definitive information regarding the substrate recognition mechanism of HtxB. However, considering the difference in the number of oxygen atoms between the Pi and HPt molecules, a difference in the number of amino acids responsible for hydrogen bond formation may account for the failure of HtxB to capture Pi. A deeper understanding of the structural basis of HtxB would help to reveal its mechanism of discriminating between Pi and Pt/HPt, as well as the minimum number of amino acid substitutions required to generate escape mutants.

As expected, RN1008, a strain lacking all endogenous P transporters and expressing PtxD and HtxABCDE, was not able to grow on media without Pt or HPt. Furthermore, we showed that this strain did not yield any escape mutants under non-permissive growth conditions with an assay detection limit of 1.9 × 10^−13^ per CFU, which is lower than that of any other reported biocontainment strategies. For the practical application of GMMs, the escape frequency should be low enough to ensure biosafety. Recent works showed that deliveries of 10^6^ to 10^9^ living GMMs into mice were effective for vaccination^34^,^35^ or oncolytic therapies^36^. The use of GMM-based vaccinations or therapeutics in humans, however, would require development of a secure biocontainment system that can exclude generation of escape mutants. Considering the escape frequency of our strategy, it could be used in applications that involve the release of a large population of cells, such as therapeutics or bioremediation. We also note that this containment efficacy was achieved using a single strategy without any additional genetic modifications for preventing the generation of escape mutants. The extremely low escape frequency of our biocontainment strategy may be due to the fundamental role of Pi in cellular metabolism. Pi is required for numerous cellular metabolic events, such as ATP synthesis, nucleic acid synthesis, and signal transduction. In particular, nucleic acid synthesis, which is involved in the generation of most types of adaptive mutations^37^, requires a considerable amount of Pi. Thus, depletion of cellular Pi may result in the stalling of most metabolic events, including the induction of genetic mutations.

The growth of RN1008 strictly depends on Pt and HPt. Although Pt and HPt are rarely detected in the environment, we should consider the possible origins of Pt and HPt in order to manage potential risks of this containment strategy. Reduced P compounds could be produced and enter the environment through several routes. First, some earlier reports proposed that microorganisms that are present in anoxic environments such as paddy fields^38^ and digested activated sludge^39^ may reduce Pi to produce Pt, HPt, and/or phosphine (PH_3_). However, these reports have never been confirmed by other works^25^. The reduction potential of the Pi/Pt couple is −650 mV, which far exceeds that of the NAD/NADH couple (−320 mV)^40^, suggesting that direct reduction of Pi by biological processes is highly improbable. Second, several geochemical reactions such as lightning strikes^41^ and volcanic eruptions^42^ could reduce Pi or release reduced P compounds. However, they occur infrequently and do not produce significant amounts of reduced P at the same locations continuously. We showed that in the absence of Pt, the viability of RN1008 cells was reduced to approximately 1.0 × 10^−4^% after seven days, and no cells were able to survive for more than 14 days (Fig. 5). Therefore, even if the Pt-dependent bacteria were to encounter these rare Pt sources along with other necessary nutrients before oxidation or consumption by indigenous bacteria, they would eventually deplete available Pt and lose viability. Third, reduced inorganic P compounds have been used as fumigants, reducing agents, and fungicides^25^,^43^,^44^. Although the former two applications are generally carried out at closed facilities, the use of Pt as fungicide in agriculture is the largest anthropogenic source of Pt in the environment^44^. In this application, Pt is used at relatively diluted levels (0.1~3 mM)^44^ but is applied directly to farmland, or plant foliage. Following Pt application, its concentration in soil may be high enough to allow survival or growth of Pt-dependent bacteria. Therefore, accidental release of Pt-dependent bacteria near Pt-treated farmland may potentially compromise the containment ability of our strategy. However, various microorganisms in soil and water rapidly take up and oxidize Pt to supplement their Pi requirement^24^,^45^. In contrast to phosphate fertilizer, even regular application of Pt does not result in its accumulation in soil, as the applied Pt is rapidly oxidized by the resident microbial community^45^,^46^. In the near future, we plan to test the survival of the Pt/HPt-dependent bacteria in soil following single or repeated application of Pt.

Although the origins and ubiquity of Pt and HPt remain unclear, several recent works have reported detection of micromolar levels of Pt and HPt in the environmental water samples from a eutrophic lake^47^ (Lake Taihu, China, ~0.45 μM), a geothermal spring^48^ (Hot Creek Gorge, CA, USA, ~0.06 μM), and a river^42^ (River Front Park, FL, USA, ~1.97 μM). We therefore performed an additional experiment to investigate whether such low concentrations of Pt could support proliferation of RN1008 and extend the viability of the strain. Approximately 5.0 × 10^6^ CFU of RN1008 was cultured in 5 mL MOPS medium containing 5 μM Pt, and its CFU on MOPS-Pt plate was monitored over 14 days. Although CFU count did not significantly change after 1 day, it dropped below 10^4^ CFU after 6 days, and became undetectable after 14 days. We found that RN1008 could neither grow nor survive in the presence of 5 μM of Pt, which exceeds the highest reported environmental concentration, suggesting that this containment strategy should be effective even in such Pt-containing environments.

Another possible risk for this strategy would be the acquisition of genes conferring Pi transport ability by the Pt/HPt-dependent strain via horizontal gene transfer (HGT). Although we did not evaluate the probability of mutant escape via HGT, the integration of a single gene with Pi transport function would be sufficient to generate escape strains. In order to reduce the above risks, the developed strategy should be used in combination with other containment strategies. The requirements for creating the containment strain are the disruption of eight genes involved in Pi uptake and introduction of the genes *ptxD* and *htxABCDE*. In contrast to more complicated biocontainment strategies that involve hundreds of genetic modifications, the developed strategy could be easily combined with other containment methodologies and applied to established host strains. In this study, we demonstrated the applicability of this strategy in *E. coli* as a model organism. However, we think that it would also be applicable to other microorganisms including Gram-positive bacteria if a reliable gene expression and disruption system were available. Recently developed genome-editing techniques for a variety of bacteria^49^,^50^ would greatly simplify the task of disrupting multiple genes for Pi-uptake, thus making it easier to create Pt-dependent strains.

In addition to technical convenience, our system offers the ease of mass cultivation of the engineered microbe. As the cost of Pt is extremely low compared to those of other commonly used selection chemicals for biocontainment, the Pt/HPt dependency of the engineered strain provides a cost-effective, antibiotic-free cultivation method^26^,^51^,^52^. Another specific advantage of our containment strategy is related to a particular characteristic of the required nutrient. Pt is non-reusable and no more present in the culture medium or even cell bodies after consumption by Pt-dependent bacteria. In contrast, other auxotrophic chemicals such as NSAAs persist in the cell body and could be released and reused after cell death, significantly extending the survival of auxotrophic cells. The non-reusable characteristic of Pt would significantly reduce the risk of the “key” nutrient persisting in the environment after an accidental release. These advantages, together with its strikingly low escape frequency, make Pt/HPt dependency a promising biocontainment strategy for practical applications.

## Methods

### Bacteria and media

Bacterial strains used in this study are listed in Supplementary Table 1. Routine culture of *E. coli* strains was conducted in 2× yeast extract-tryptone (2xYT) medium. Morpholinepropanesulfonic acid-glucose synthetic (MOPS) medium^53^ was used as a minimal medium. MOPS media containing Pi, Pt, HPt, and glycerol 3-phosphate (G3Pi) are designated as MOPS-Pi, MOPS-Pt, MOPS-HPt, and MOPS-G3Pi, respectively. P-free MOPS medium is designated as MOPS-0. The P concentration of MOPS media was 1.0 mM unless otherwise stated. Pt and HPt stock solutions (1.0 M) were prepared by dissolving phosphorous acid (Nakarai Tesque, Kyoto, Japan) or sodium hypophosphite in distilled water, respectively. These solutions were neutralized with sodium hydroxide, filtered, aliquoted, and stored at −20 °C until use. Soil extract (SE) was prepared from humic soils purchased at a local market. Briefly, 0.1 kg air-dried soil was mixed with 0.3 L of tap water and autoclaved for 30 min. The extract was serially filtered with Whatman 3MM paper and a 0.45-μm pore-sized filter membrane to remove insoluble matter. Two SEs (SE[A] and SE[B]) were prepared from two different humic soils. Inorganic Pi contents in SEs were measured by the molybdenum blue method^54^. Total P concentration (T-P) was assessed as Pi after ammonium persulfate digestion at 121 °C for 30 min^54^. P concentration other than inorganic Pi (various organic P compounds such as inositol Pi or polyphosphates) can be determined by subtracting the inorganic Pi concentration from T-P concentration. Sheep blood agar plates (Kohjin BIO, Saitama, Japan), 2xYT, Terrific Broth (BD Biosciences, Franklin Lakes, NJ, USA), and chocolate agar plates (BD Biosciences) were used for challenges with naturally available P sources. The concentrations of antibiotics used were 50 mg/L for ampicillin or kanamycin and 25 mg/L for chloramphenicol.

### Plasmid construction

Plasmids and primers used in this study are listed in Supplementary Table 2. The gene coding for PtxABC of *Ralstonia* sp. 4506^55^ (accession number: LC187312) was amplified by PCR from genomic DNA using primer pair EcoPtxA(−186)_fw/BamPtxC(+13)_rv. The resultant PCR fragment was inserted into the *Eco*RI/*Bam*HI cloning site of pMW118. The DNA fragment containing *htxABCDE* of *P. stutzeri* WM88 (accession number: AF061267) was amplified from genomic DNA using primer pair htxA-14_fw2/htxE_rv2 and was inserted into the *Bam*HI sites of pMW118 and pSTV28 by using the In-Fusion HD cloning kit (Takara Bio, Inc., Tokyo, Japan). For construction of ptxD/pSTV28, a DNA fragment containing *ptxD* was amplified from *Ralstonia* sp. 4506 genomic DNA using primer pair EcoPtxD(−157)_fw/BamHI-ptxD(+24)_rv and was introduced into the *Eco*RI/*Bam*HI site of pSTV28. For construction of Ptac4071- and Ptac4073-ptxD/pTWV229, primer pair ptxD_fw/ptxD_rv was used to amplify the *ptxD* gene from *Ralstonia* sp. 4506. The amplified DNA fragment was ligated to pTWV229ΔPlac-Ptac4071 and pTWV229ΔPlac-Ptac4073^56^, which was linearized by PCR using primer pair Ptac4071-fw/Ptac4071-rv, by the In-Fusion HD cloning method. These plasmids express PtxD with a 15-amino acid sequence extension (ARHKLPGIKLSRRPS) at the C-terminus end, which increases PtxD activity approximately 6.8-fold of that of wild-type protein in *E. coli* MG1655. All DNA fragments cloned into vector plasmids were verified by sequencing.

### Characterization of P transporters

The pMW118-derivative plasmids carrying genes for *ptxABC* or *htxABCDE* were individually introduced into the Pi transporter-null mutant MT2012^28^ expressing PtxD (MT2012-*ptxD*). For the growth assay, cells grown on MOPS-G3Pi were collected by centrifugation, washed three times with MOPS-0, and resuspended to an optical density at 600 nm (OD_600_) of 1.0. Next, 50 μL of each cell suspension was used to inoculate glass test tubes containing 5 mL MOPS-Pi or MOPS-Pt, and these cultures were incubated at 37 °C with constant shaking (160 rpm). The turbidities of the cultures were monitored using an OD monitoring instrument (OD-Monitor C&T; Taitec Co. Ltd., Saitama, Japan).

### Pi incorporation analysis

[^32^P]-labeled Pi ([^32^P]Pi) uptake by *E. coli* cells was carried out under conditions in which Pi uptake was linear over time. Cells grown to mid-log phase in MOPS-G3Pi medium at 37 °C were collected by centrifugation, washed two times in MOPS-0, and resuspended in MOPS-0 to an OD_600_ of 0.2. Cells were kept at 37 °C until the assay was performed. Each assay was started by adding 200 μL of cell suspension to 1 mL of pre-warmed MOPS medium containing 20 μM Pi and [^32^P]Pi (MP Biomedicals, Santa Ana, CA, USA). After the addition of the [^32^P]Pi solution, a 200-μL aliquot was removed at various times, filtered, and the filter was washed twice with 5 mL of an ice-cold 10 mM LiCl solution. The filter was then transferred to a scintillation vial containing 5 mL of scintillation cocktail (GE Healthcare, Marlborough, MA, USA), and the amount of radioactivity taken up by cells was measured with a liquid scintillation counter.

### Engineering of P metabolic pathway

Figure 1b illustrates the strategy used to construct an *E. coli* recombinant whose growth is strictly dependent on Pt/HPt. To construct a mutant deficient in Pi and organic Pi transport, three organic Pi transporters (*glpT, ugpB*, and *uhpT*) were disrupted in addition to four Pi transporters (*pitA, pitB, phnCDE*, and *pstSCAB*)^57^. The gene for *phoA* was also disrupted because *E. coli* alkaline phosphatase exhibits weak Pt-oxidizing activity^58^ which could result in oxidization of Pt in the periplasmic space, reducing the amount of Pt taken up. At the beginning of the construction scheme, deletion of the organic Pi transporter *glpT* from the *E. coli* Keio collection^59^ (National BioResource Project, National Institute of Genetics, Shizuoka, Japan) was transferred to the *E. coli* mutant MT2010 (∆*pitA*, ∆*pitB*, ∆*phnC*, and ∆*phoA*)^28^ by P1 transduction. A kanamycin (Kan) resistance cassette in the chromosome of the resultant strain was eliminated by flippase (FLP)-mediated recombination using the pCP20 plasmid^60^. After plasmid clearance of the Kan-sensitive clone, which we designated RN1002, *ugpB* and *uhpT* were sequentially disrupted in the same manner. The resultant strain, lacking all P transporters except PstSCAB, was designated RN1006. Before disruption of *pstSCAB*, htxABCDE/pSTV28 and Ptac4071-ptxD/pTWV229 were simultaneously introduced into RN1006 to support the uptake and subsequent oxidation of Pt, resulting in strain RN1007. We used these two plasmids to balance the expression levels of PtxD and HtxABCDE, since we observed that high expression level of PtxD was required to support faster growth in MOPS-Pt, whereas excessive expression of HtxABCDE was detrimental to the cell growth (Supplementary Fig. 1). Disruption of *pstSCAB* was completed last because the growth of *E. coli* using Pi as a P source is faster than that of cells using organic Pi compounds, facilitating cell culture during the series of gene disruption experiments. *phoU*, the last gene in the *pst* operon, was deleted along with *pstSCAB* because inactivation of the *pst* gene results in severe defects in cell growth and generates various back mutants, probably due to the unknown function of the sensor protein PhoU^61^,^62^. P1 lysate prepared from BW17355 was transduced into RN1007, and cells were incubated at 37 °C for 3 hours after the medium was replaced with MOPS-Pt. This cell suspension was plated on MOPS-Pt agar plates containing ampicillin, chloramphenicol, and kanamycin, and the resultant strain, RN1008, lacking all seven endogenous P transporters, was obtained. RN1008 could also be propagated on MOPS-Pt plates without antibiotics due to the selective pressure of Pt availability. Gene disruption was confirmed by PCR.

### Environmental challenges and escape assay

To determine whether RN1008 was able to grow on other Pi sources, a spot assay was performed in which 1 mL of RN1008 culture grown on MOPS-Pt at late log phase was centrifuged, washed once with an equal volume of MOPS-0, and diluted using a 10-fold series by 10^7^-fold. Next, 10 μL of each diluted aliquot was spotted onto various culture media plates including Luria-Bertani (LB), 2xYT, Terrific Broth, sheep blood agar and chocolate agar plates, which were incubated at 37 °C, and the growth was monitored for 7 days. In order to investigate escape mutant generation, 5-L of RN1008 culture was grown to late log phase, pelleted, washed once with 100 mL MOPS-0, and resuspended in 10 mL MOPS-0. This cell suspension was plated on 50 large, square dish plates (245 mm × 245 mm × 25 mm; ThermoFisher Scientific, Kanagawa, Japan) containing 2xYT agar medium, and colony formation was monitored for 21 days. Aliquots of the culture were diluted, spread onto MOPS-Pt plates, and CFU were counted after 48 h of incubation at 37 °C in order to determine total cell numbers used for the assay. The detection limit was calculated as one per the total CFU plated. For the viability assay, 1 mL of an overnight culture of RN1008 in MOPS-Pt medium was pelleted by centrifugation, washed once with MOPS-0, and resuspended in MOPS-0 to an OD_600_ of approximately 1.0. Then, 1 mL of the cell suspension was added to a 500-mL Erlenmeyer flask containing 100 mL of MOPS-Pt or 2xYT medium. At each sampling point, 0.5 mL of culture was collected from each flask, and its OD_600_ was measured. It was then diluted with MOPS-0 and spread onto 2xYT or MOPS-Pt agar plates. Dilution rates for spreading onto (permissive) MOPS-Pt plates ranged from 10^3^-fold to 10^6^-fold; for spreading onto (non-permissive) 2xYT plates, 0.1 mL of undiluted culture was used directly. Data were collected for 14 days following inoculation.

## Additional Information

**How to cite this article**: Hirota, R. *et al*. A Novel Biocontainment Strategy Makes Bacterial Growth and Survival Dependent on Phosphite. *Sci. Rep.* **7**, 44748; doi: 10.1038/srep44748 (2017).

**Publisher's note:** Springer Nature remains neutral with regard to jurisdictional claims in published maps and institutional affiliations.

## Supplementary Material

Supplementary Information

## References

[b1] Nielsen J. & Keasling J. D. Engineering Cellular Metabolism. Cell 164, 1185–1197, doi: 10.1016/j.cell.2016.02.004 (2016). 26967285

[b2] Stephanopoulos G. Synthetic biology and metabolic engineering. ACS Synth Biol 1, 514–525, doi: 10.1021/sb300094q (2012). 23656228

[b3] Baker M. Synthetic genomes: The next step for the synthetic genome. Nature 473, 403, 405–408, doi: 10.1038/473403a (2011). 21593873

[b4] Ostrov N. . Design, synthesis, and testing toward a 57-codon genome. Science 353, 819–822, doi: 10.1126/science.aaf3639 (2016). 27540174

[b5] Wright O., Delmans M., Stan G. B. & Ellis T. GeneGuard: A modular plasmid system designed for biosafety. ACS Synth. Biol. 4, 307–316, doi: 10.1021/sb500234s (2015). 24847673

[b6] Pinero-Lambea C., Ruano-Gallego D. & Fernandez L. A. Engineered bacteria as therapeutic agents. Curr. Opin. Biotechnol. 35, 94–102, doi: 10.1016/j.copbio.2015.05.004 (2015). 26070111

[b7] Berg P., Baltimore D., Brenner S., Roblin R. O. & Singer M. F. Summary statement of the Asilomar conference on recombinant DNA molecules. Proc. Natl. Acad. Sci. USA 72, 1981–1984 (1975). 806076 10.1073/pnas.72.6.1981 PMC432675

[b8] Molin S. . Suicidal genetic elements and their use in biological containment of bacteria. Ann. Rev. Microbiol. 47, 139–166, doi: 10.1146/annurev.mi.47.100193.001035 (1993). 8257096

[b9] Caliando B. J. & Voigt C. A. Targeted DNA degradation using a CRISPR device stably carried in the host genome. Nat. Commun. 6, 6989, doi: 10.1038/ncomms7989 (2015). 25988366 PMC4479009

[b10] Curtiss R. 3rd. Biological containment and cloning vector transmissibility. J. Infect. Dis. 137, 668–675 (1978). 351084 10.1093/infdis/137.5.668

[b11] Ronchel M. C. & Ramos J. L. Dual system to reinforce biological containment of recombinant bacteria designed for rhizoremediation. Appl. Environ. Microbiol. 67, 2649–2656, doi: 10.1128/AEM.67.6.2649-2656.2001 (2001). 11375176 PMC92920

[b12] Lopez G. & Anderson J. C. Synthetic auxotrophs with ligand-dependent essential genes for a BL21(DE3) biosafety strain. ACS Synth. Biol. 4, 1279–1286, doi: 10.1021/acssynbio.5b00085 (2015). 26072987

[b13] Mandell D. J. . Biocontainment of genetically modified organisms by synthetic protein design. Nature 518, 55–60, doi: 10.1038/nature14121 (2015). 25607366 PMC4422498

[b14] Rovner A. J. . Recoded organisms engineered to depend on synthetic amino acids. Nature 518, 89–93, doi: 10.1038/nature14095 (2015). 25607356 PMC4590768

[b15] Gallagher R. R., Patel J. R., Interiano A. L., Rovner A. J. & Isaacs F. J. Multilayered genetic safeguards limit growth of microorganisms to defined environments. Nucleic Acids Res. 43, 1945–1954, doi: 10.1093/nar/gku1378 (2015). 25567985 PMC4330353

[b16] Hoang T. T., Williams S., Schweizer H. P. & Lam J. S. Molecular genetic analysis of the region containing the essential *Pseudomonas aeruginosa asd* gene encoding aspartate-beta-semialdehyde dehydrogenase. Microbiology 143 (Pt 3), 899–907, doi: 10.1099/00221287-143-3-899 (1997). 9084174

[b17] Metcalf W. W. & Wolfe R. S. Molecular genetic analysis of phosphite and hypophosphite oxidation by *Pseudomonas stutzeri* WM88. J. Bacteriol. 180, 5547–5558 (1998). 9791102 10.1128/jb.180.21.5547-5558.1998 PMC107611

[b18] White A. K. & Metcalf W. W. Isolation and biochemical characterization of hypophosphite/2-oxoglutarate dioxygenase. A novel phosphorus-oxidizing enzyme from *Pseuedomonas stutzeri* WM88. J. Biol. Chem. 277, 38262–38271, doi: 10.1074/jbc.M204605200 (2002). 12161433

[b19] Costas A. M., White A. K. & Metcalf W. W. Purification and characterization of a novel phosphorus-oxidizing enzyme from *Pseudomonas stutzeri* WM88. J. Biol. Chem. 276, 17429–17436, doi: 10.1074/jbc.M011764200 (2001). 11278981

[b20] Karl D. M. Microbially mediated transformations of phosphorus in the sea: new views of an old cycle. Ann. Rev. Mar. Sci. 6, 279–337, doi: 10.1146/annurev-marine-010213-135046 (2014). 24405427

[b21] Metcalf W. W. . Synthesis of methylphosphonic acid by marine microbes: a source for methane in the aerobic ocean. Science 337, 1104–1107, doi: 10.1126/science.1219875 (2012). 22936780 PMC3466329

[b22] Van Mooy B. A. . Phosphorus cycling. Major role of planktonic phosphate reduction in the marine phosphorus redox cycle. Science 348, 783–785, doi: 10.1126/science.aaa8181 (2015). 25977548

[b23] Yu X. . Diversity and abundance of phosphonate biosynthetic genes in nature. Proc. Natl. Acad. Sci. USA 110, 20759–20764, doi: 10.1073/pnas.1315107110 (2013). 24297932 PMC3870699

[b24] White A. K. & Metcalf W. W. Microbial metabolism of reduced phosphorus compounds. Annu. Rev. Microbiol. 61, 379–400, doi: 10.1146/annurev.micro.61.080706.093357 (2007). 18035609

[b25] Morton S. C. & Edwards M. Reduced Phosphorus Compounds in the Environment. Crit. Rev. Environ. Sci. Technol. 35, 333–364, doi: 10.1080/10643380590944978 (2005).

[b26] Kanda K. . Application of a phosphite dehydrogenase gene as a novel dominant selection marker for yeasts. J. Biotechnol. 182–183, 68–73, doi: 10.1016/j.jbiotec.2014.04.012 (2014). 24786825

[b27] Zhao H. & van der Donk W. A. Regeneration of cofactors for use in biocatalysis. Curr. Opin. Biotechnol. 14, 583–589, doi: 10.1016/j.copbio.2003.09.007 (2003). 14662386

[b28] Motomura K. . Overproduction of YjbB reduces the level of polyphosphate in *Escherichia coli*: a hypothetical role of YjbB in phosphate export and polyphosphate accumulation. FEMS Microbiol. Lett. 320, 25–32, doi: 10.1111/j.1574-6968.2011.02285.x (2011). 21488939

[b29] Sprague G. F. Jr., Bell R. M. & Cronan J. E. Jr. A mutant of *Escherichia coli* auxotrophic for organic phosphates: evidence for two defects in inorganic phosphate transport. Mol Gen Genet 143, 71–77 (1975). 765745 10.1007/BF00269422

[b30] Ju K. S. . Discovery of phosphonic acid natural products by mining the genomes of 10,000 actinomycetes. Proc. Natl. Acad. Sci. USA 112, 12175–12180, doi: 10.1073/pnas.1500873112 (2015). 26324907 PMC4593130

[b31] White A. K. & Metcalf W. W. The *htx* and *ptx* operons of *Pseudomonas stutzeri* WM88 are new members of the *pho* regulon. J. Bacteriol. 186, 5876–5882, doi: 10.1128/JB.186.17.5876-5882.2004 (2004). 15317793 PMC516845

[b32] Liebschner D. . Elucidation of the phosphate binding mode of DING proteins revealed by subangstrom X-ray crystallography. J. Am. Chem. Soc. 131, 7879–7886, doi: 10.1021/ja901900y (2009). 19445459

[b33] Neznansky A., Blus-Kadosh I., Yerushalmi G., Banin E. & Opatowsky Y. The *Pseudomonas aeruginosa* phosphate transport protein PstS plays a phosphate-independent role in biofilm formation. FASEB J. 28, 5223–5233, doi: 10.1096/fj.14-258293 (2014). 25223609

[b34] Garmory H. S., Griffin K. F., Brown K. A. & Titball R. W. Oral immunisation with live *aroA* attenuated *Salmonella enterica* serovar Typhimurium expressing the *Yersinia pestis* V antigen protects mice against plague. Vaccine 21, 3051–3057 (2003). 12798649 10.1016/s0264-410x(03)00112-9

[b35] Kang H. Y., Srinivasan J. & Curtiss R. 3rd. Immune responses to recombinant pneumococcal PspA antigen delivered by live attenuated *Salmonella enterica* serovar typhimurium vaccine. Infect. Immun. 70, 1739–1749 (2002). 11895935 10.1128/IAI.70.4.1739-1749.2002 PMC127874

[b36] Anderson J. C., Clarke E. J., Arkin A. P. & Voigt C. A. Environmentally controlled invasion of cancer cells by engineered bacteria. J. Mol. Biol. 355, 619–627, doi: 10.1016/j.jmb.2005.10.076 (2006). 16330045

[b37] Foster P. L. Adaptive mutation: the uses of adversity. Ann. Rev. Microbiol. 47, 467–504, doi: 10.1146/annurev.mi.47.100193.002343 (1993). 8257106 PMC2989722

[b38] Tsubota G. Phosphate reduction in the paddy field I. Soil Sci. Plant Nutr. 5, 10–15, doi: 10.1080/00380768.1959.10430888 (1959).

[b39] Dévai I., Felföldy L., Wittner I. & Plósz S. Detection of phosphine: new aspects of the phosphorus cycle in the hydrosphere. Nature 333, 343–345, doi: 10.1038/333343a0 (1988).

[b40] Roels J. & Verstraete W. Biological formation of volatile phosphorus compounds. Bioresour. Technol. 79, 243–250 (2001). 11499578 10.1016/s0960-8524(01)00032-3

[b41] Pasek M. & Kristin B. Lightning-induced reduction of phosphorus oxidation state. Nat. Geosci. 2, 553–556 (2009).

[b42] Pasek M. A., Sampson J. M. & Atlas Z. Redox chemistry in the phosphorus biogeochemical cycle. Proc. Natl. Acad. Sci. USA 111, 15468–15473, doi: 10.1073/pnas.1408134111 (2014). 25313061 PMC4217446

[b43] Hanrahan G., Salmassi T. M., Khachikian C. S. & Foster K. L. Reduced inorganic phosphorus in the natural environment: significance, speciation and determination. Talanta 66, 435–444, doi: S0039-9140(04)00620-4 [pii] 10.1016/j.talanta.2004.10.004 (2005). 18970004

[b44] McDonald A. E., Grant B. R. & Plaxton W. C. Phosphite (phosphorous acid): Its relevance in the environment and agriculture and influence on plant phosphate starvation response. J. Plant Nutr. 24, 1505–1519, doi: 10.1081/Pln-100106017 (2001).

[b45] Adams F. & Conrad J. P. Transition of phosphite to phosphate in soils. Soil Science 75, 361–371 (1953).

[b46] Stöven K., Schroetter S., Panten K. & Schnug E. Effect of phosphite on soil microbial enzyme activity and the feeding activity of soil mesofauna. Landbauforschung Völkenrode 57, 127–131 (2007).

[b47] Han C. . Phosphite in sedimentary interstitial water of Lake Taihu, a large eutrophic shallow lake in China. Environ. Sci. Technol. 47, 5679–5685, doi: 10.1021/es305297y (2013). 23647420

[b48] Pech H. . Detection of geothermal phosphite using high-performance liquid chromatography. Environ. Sci. Technol. 43, 7671–7675, doi: 10.1021/es901469t (2009). 19921877 PMC2780435

[b49] Oh J. H. & van Pijkeren J. P. CRISPR-Cas9-assisted recombineering in *Lactobacillus reuteri*. Nucleic Acids Res. 42, e131, doi: 10.1093/nar/gku623 (2014). 25074379 PMC4176153

[b50] Wendt K. E., Ungerer J., Cobb R. E., Zhao H. & Pakrasi H. B. CRISPR/Cas9 mediated targeted mutagenesis of the fast growing cyanobacterium *Synechococcus elongatus* UTEX 2973. Microb. Cell Fact. 15, 115, doi: 10.1186/s12934-016-0514-7 (2016). 27339038 PMC4917971

[b51] Loera-Quezada M. M. . A novel genetic engineering platform for the effective management of biological contaminants for the production of microalgae. Plant Biotechnol. J. 14, 2066–2076, doi: 10.1111/pbi.12564 (2016). 27007496 PMC5043480

[b52] Shaw A. J. . Metabolic engineering of microbial competitive advantage for industrial fermentation processes. Science 353, 583–586, doi: 10.1126/science.aaf6159 (2016). 27493184

[b53] Neidhardt F. C., Bloch P. L. & Smith D. F. Culture medium for enterobacteria. J. Bacteriol. 119, 736–747 (1974). 4604283 10.1128/jb.119.3.736-747.1974 PMC245675

[b54] Kuroda A. . A simple method to release polyphosphate from activated sludge for phosphorus reuse and recycling. Biotechnol. Bioeng. 78, 333–338 (2002). 11920449 10.1002/bit.10205

[b55] Hirota R. . Isolation and characterization of a soluble and thermostable phosphite dehydrogenase from *Ralstonia* sp. strain 4506. J. Biosci. Bioeng. 113, 445–450, doi: 10.1016/j.jbiosc.2011.11.027 (2012). 22197497

[b56] Abe K., Kuroda A. & Takeshita R. Engineering of *Escherichia coli* to facilitate efficient utilization of isomaltose and panose in industrial glucose feedstock. Appl. Microbiol. Biotechnol. in press (2016). 10.1007/s00253-016-8037-z PMC5309279 27933453

[b57] Hirota R., Kuroda A., Kato J. & Ohtake H. Bacterial phosphate metabolism and its application to phosphorus recovery and industrial bioprocesses. J. Biosci. Bioeng. 109, 423–432, doi: 10.1016/j.jbiosc.2009.10.018 (2010). 20347763

[b58] Yang K. & Metcalf W. W. A new activity for an old enzyme: *Escherichia coli* bacterial alkaline phosphatase is a phosphite-dependent hydrogenase. Proc. Natl. Acad. Sci. USA 101, 7919–7924, doi: 10.1073/pnas.0400664101 (2004). 15148399 PMC419532

[b59] Baba T. . Construction of *Escherichia coli* K-12 in-frame, single-gene knockout mutants: the Keio collection. Mol. Syst. Biol. 2, 2006 0008, doi: 10.1038/msb4100050 (2006). PMC1681482 16738554

[b60] Datsenko K. A. & Wanner B. L. One-step inactivation of chromosomal genes in *Escherichia coli* K-12 using PCR products. Proc. Natl. Acad. Sci. USA 97, 6640–6645, doi: 10.1073/pnas.120163297 (2000). 10829079 PMC18686

[b61] Hirota R. . Stable polyphosphate accumulation by a pseudo-revertant of an *Escherichia coli phoU* mutant. Biotechnol. Lett. 35, 695–701, doi: 10.1007/s10529-012-1133-y (2013). 23288295

[b62] Steed P. M. & Wanner B. L. Use of the *rep* technique for allele replacement to construct mutants with deletions of the *pstSCAB-phoU* operon: evidence of a new role for the PhoU protein in the phosphate regulon. J. Bacteriol. 175, 6797–6809 (1993). 8226621 10.1128/jb.175.21.6797-6809.1993 PMC206803

