# Supplementary material for: A Novel Biocontainment Strategy Makes Bacterial Growth and Survival Dependent on Phosphite
Source: Sci Rep. 2017 Mar 20;7:44748. doi: 10.1038/srep44748 (PMC5357788; doi:10.1038/srep44748)
Supplement: Supplementary Information [file srep44748-s1.pdf]

Supplementary information

A Novel Biocontainment Strategy Makes Bacterial Growth and Survival Dependent on Phosphite

Ryuichi HIROTA, Kenji ABE, Zen-ichiro KATSUURA, Reiji NOGUCHI, Shigeaki MORIBE, Kei MOTOMURA, Takenori ISHIDA, Maxym ALEXANDROV, Hisakage FUNABASHI, Takeshi IKEDA, Akio KURODA

Table of Contents

Supplementary Table 1. Bacterial strains used in this study.

Supplementary Table 2. Primers and plasmids used in this study.

Supplementary Figure 1. Choices of the expression system for PtxD and HtxABCDE.

Supplementary Table 1. Bacterial strains used in this study

Strain	Description	Reference or source
E. coli		
DH5α	Cloning host strain	Toyobo Co. Ltd.
MG1655	Wild-type strain; F^- *arcA-1655 fnr-1655*	Laboratory stock
BW25113	*rrnB3* Δ*lacZ4787* *hsdR514* Δ(*araBAD*)*567* Δ(*rhaBAD*)*568 rph-1*	60
BW17335	DE3(*lac*)X74 Δ(*pstSCAB-phoU*)*560*::Km^r	62
JW2234	BW25113 Δ*glpT*::Km^r	NBRP
JW3418	BW25113 Δ*ugpB*::Km^r	NBRP
JW3641	BW25113 Δ*uhpT*::Km^r	NBRP
MT2010	MG1655 Δ*pitA*::*frt* Δ*pitB*::*frt* Δ*phnC*::*frt* Δ*phoA::frt*	28
MT2012	MG1655 Δ*pitA*::*frt* Δ*pitB*::*frt* Δ*phnC*::*frt* *phoA::frt* *pstSCABphoU::kan*	28
MT2012-ptxD	MT2012 harboring ptxD/pSTV	
RN1002	MG1655 Δ*pitA*::*frt* Δ*pitB*::*frt* Δ*phnC*::*frt* *phoA::frt* Δ*glpT::frt*	This study
RN1004	MG1655 Δ*pitA*::*frt* Δ*pitB*::*frt* Δ*phnC*::*frt* *phoA::frt* Δ*glpT::frt* Δ*ugpB::frt*	This study
RN1006	MG1655 Δ*pitA*::*frt* Δ*pitB*::*frt* Δ*phnC*::*frt* *phoA::frt* Δ*glpT::frt* Δ*ugpB::frt* Δ*uhpT::frt*	This study
RN1007	MG1655 Δ*pitA*::*frt* Δ*pitB*::*frt* Δ*phnC*::*frt* *phoA::frt* Δ*glpT::frt* Δ*ugpB::frt* Δ*uhpT::frt* Ptac4071-ptxD/pTWV229, htxABCDE/pSTV28	This study
RN1008	MG1655 Δ*pitA*::*frt* Δ*pitB*::*frt* Δ*phnC*::*frt* *phoA::frt* Δ*glpT::frt* Δ*ugpB::frt* Δ*uhpT::frt* Δ(*pstSCAB-phoU*)*560*::Km^r, Ptac4071-ptxD/pTWV229, htxABCDE/pSTV28, Pt/HPt-dependent strain	This study

Ralstonia sp. 4506	Pt-oxidizer, harboring *ptxABCD* gene	57
Pseudomonas stutzeri WM88	HPt- and Pt-oxidizer, harboring *htxABCDE* gene	17

Supplementary Table 2. Primers and plasmids used in this study

Name	Sequence (5'-3') or description	Source
**Primers[1]**		
EcoPtxA(-186)_fw	aagaattcTAGCAGGCGTCTATATTTGGCATAG	-
BamPtxC(+13)_rv	aaatctagaGCTTTGGGAGTTATTTGAACTTGCG	-
EcoPtxD(-157)_fw	aagaattcAATCGGGTTCGAGCTGATGGGCTC	-
BamPtxD(+24)_rv	aaatctagaTCGCCACACGCTCCAGATCTATCAC	-
htxA-14_fw2	cggtacccggggatcCTAGGAGCATCACCATGTTTGCAGAGC	-
htxE_rv2	cgactctagaggatcTCAGATCAGCTTGGCGCGGATGCGCGCCTG	-
Ptac4071-fw	GCCCGCCATAAACTGCCAGGCATC	-
Ptac4071-rv	GGCAGTCTCCTTGTGTGAAATTGTTATCCG	-
ptxD-fw	cacaaggagactgccATGAAGCCCAAAGTCGTCCTC	-
ptxD-rv	cagtttatggcgggcCGCCGCCTTTACTCCCGGATAC	-
pitA_chkx1-fw	CGTTGCGCTCCTCTTAGAAAA	-
pitA_chkx3-rv	GTGTTAACTGATTGGCAGCG	-
pitB_chkx1-fw	TTAACCAGTGGAATACCTGTG	-
pitB_chkx3-rv	CTCAGAATATCCGTTCAACC	-
phnC_chkx1-fw	AACTGTTCCGACGCGATTGC	-
phnC_chkx2-rv	ATCAACACGCTCCAGTAACC	-
pstSCABphoU_chk-fw	GACGTCGAAATCGCCTCTGAATTCC	-
pstSCABphoU_chk-rv	ACTTCAGATGTGTAACCAGTCGCTG	-
phoA_chkx1-fw	GAGTCGAAAGAACTGTGTGC	-
phoA_chkx2-rv	GAGGAGTTAAAGGAGGTTCC	-

glpT_chk-fw	TCATAAATAAGACCACGGGC	-
glpT _chkx3-rv	ACCAGTTTATTCTGCTGAGC	-
ugpB_chk-fw	CGCATTCGGTACAACAAGAG	-
ugpB _chkx3-rv	GAAGAACAGGAAGTTGTAGC	-
uhpT_chk-fw	ACAATGCATGCCTCACGCAG	-
uhpT _chkx3-rv	CATATGGCAACACCATTGCC	-
**Plasmids**		
pMW118	Cloning vector; Amp^r, a pSC101 derivative low-copy -number vector	Nippon Gene Co. Ltd.
ptxABC/pMW118	PtxABC expression plasmid	This study
htxABCDE/pMW118	HtxABCDE expression plasmid	This study
pSTV28	Cloning vector; Cm^r, a pACYC184 derivative medium-copy-number vector	Takara Bio Inc.
ptxD/pSTV28	PtxD expression plasmid	This study
htxABCDE/pSTV28	HtxABCDE expression plasmid	This study
pTWV229ΔPlac-Ptac 4071	Cloning vector; a pTWV229 derivative medium-copy-number vector containing a *tac* promoter variant Ptac4071 in the *SmaI* site of the multi-cloning site	57
pTWV229ΔPlac-Ptac 4073	Cloning vector; a pTWV229 derivative medium-copy-number vector containing a *tac* promoter variant Ptac4073 in the *SmaI* site of the multi-cloning site	57
Ptac4071-ptxD/pTWV 229	PtxD expression plasmid under the control of Ptac4071 promoter. A 15-amino acid sequence extension that increases PtxD activity was added at the C-terminus end of wild-type PtxD.	This study
Ptac4073-ptxD/pTWV 229	PtxD expression plasmid under the control of Ptac4073 promoter. A 15-amino acid sequence extension that increases PtxD activity was added at the C-terminus end of wild-type PtxD.	This study
pCP20	FLP expression plasmid; Amp^r, temperature-sensitive replication and FLP synthesis	61

[1] Lowercase letters denote additional sequences for restriction enzyme sites. Lowercase underlined letters denote 15-bp sequences required for recombination by In-Fusion cloning reaction.

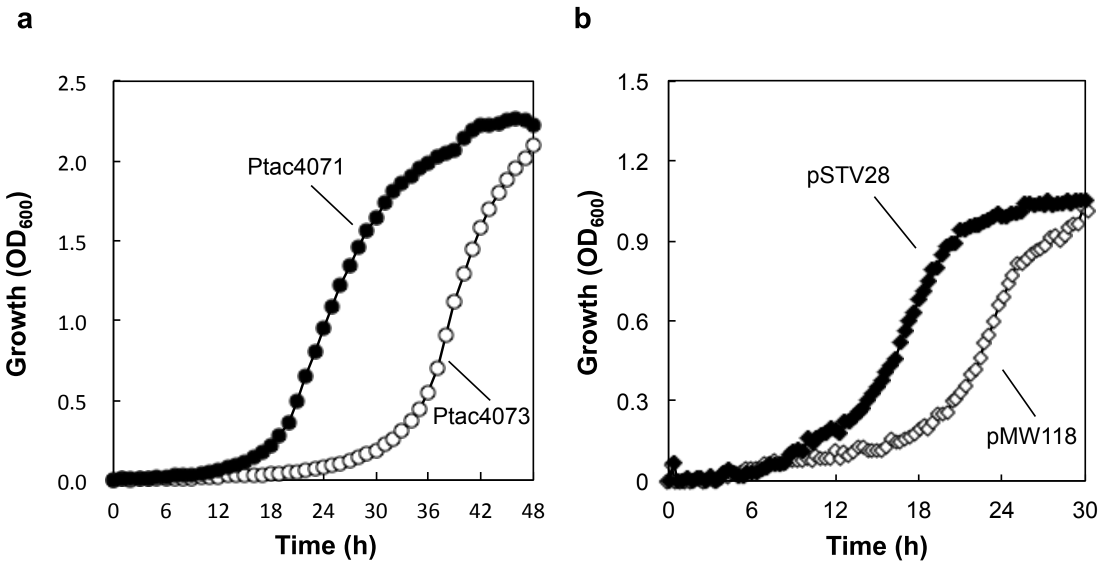

Supplementary Fig. 1. Choices of the expression system for PtxD and HtxABCDE. ***a***. The effect of promoter strength on the growth of PtxD-expressing *E. coli* in MOPS-Pt medium. *E. coli* MG1655 was transformed with the plasmids Ptac4071-ptxD/pTWV229 (closed circles) and Ptac4073-ptxD/pTWV229 (open circles), and their growth on MOPS-Pt was monitored every ten minutes. *E. coli* expressing PtxD under Ptac4071 promoter showed higher growth rate than that using Ptac4073. Since the promoter strength of Ptac4071 is approximately five times higher than that of Ptac4073, this result indicated that increased PtxD expression level could support higher growth rate of *E. coli* in MOPS-Pt. ***b***. The effect of plasmid copy numbers on the growth of HtxABCDE-expressing *E. coli* on MOPS-HPt medium. *E. coli* harboring Ptac4071-ptxD/pTWV229 was transformed with the plasmids containing *htxABCDE* gene (htxABCDE/pSTV28, closed diamonds; htxABCDE/pMW118, open diamonds). Use of the pSTV expression system resulted in faster growth than that obtained with the pMW expression system in MOPS-HPt. We failed to construct an HtxABCDE expression plasmid using a high-copy number plasmid pUC119, indicating that high expression of HtxABCDE is detrimental to the cell growth.
